# Mechanism by Which MC Controls Harmful Algal Blooms Revealed by Cell Morphology of *Aureococcus anophagefferens*

**DOI:** 10.3390/ijerph182111191

**Published:** 2021-10-25

**Authors:** Jianan Zhu, Zhiming Yu, Liyan He, Xihua Cao, Hena Ji, Xiuxian Song

**Affiliations:** 1 CAS Key Laboratory of Marine Ecology and Environmental Sciences, Institute of Oceanology, Chinese Academy of Sciences, Qingdao 266071, China; sduzjn@163.com (J.Z.); hely@qdio.ac.cn (L.H.); caoxh@qdio.ac.cn (X.C.); hena.ji@outlook.com (H.J.); songxx@qdio.ac.cn (X.S.); 2 Functional Laboratory of Marine Ecology and Environmental Science, Qingdao National Laboratory for Marine Science and Technology, Qingdao 266237, China; 3 Center for Ocean Mega-Science, Chinese Academy of Sciences, Qingdao 266071, China; 4 University of Chinese Academy of Sciences, Beijing 100049, China

**Keywords:** modified clay, harmful algal blooms, residual cells, *Aureococcus anophagefferens*, cell morphology

## Abstract

On the basis of field experience, a bloom does not continue after treatment with modified clay (MC), even though the residual harmful algal bloom (HAB) biomass accounts for 20–30% of the initial cells. This interesting phenomenon indicates that, in addition to causing flocculation, MC can inhibit the growth of residual cells. Here, from a cell morphology perspective, *Aureococcus anophagefferens* was used as a model organism to explore this scientific issue and clarify the mechanism by which MC mitigates harmful algal blooms (HABs). The results showed that, at an ~70% removal efficiency, neutral clay (NC) could not effectively inhibit the growth of residual cells, although it caused various forms of damage to residual cells, such as cell deformation, cell breakage, decreased extracellular polysaccharides (EPS), increased cell membrane permeability, and increased cytoplasmic granularity, due to physical collisions. After modification, some physical and chemical properties of the clay particle surface were changed; for example, the surface electrical properties changed from negative to positive, lamellar spacing increased, hardness decreased, adhesion chains increased, adhesion improved, and the number of absorption sites increased, enhancing the occurrence of chemical and electrochemical effects and physical collisions with residual cells, leading to severe cell deformation and chemical cell breakage. Thus, MC effectively inhibited the growth of residual cells and controlled HABs.

## 1. Introduction

Harmful algal blooms (HABs) occur when colonies of algae that live in the sea and freshwater grow out of control and produce toxic or harmful effects on people, fish, shellfish, marine mammals, and birds [1]. HABs are a global disaster, and their frequency, scale, and the number of responsible species have increased dramatically, causing profound and deleterious effects on aquatic ecosystems, aquaculture, tourism, and public health [2,3,4].

Due to these adverse effects, countries and regions with frequent HABs have explored several disposal methods. Clay-flocculating algal organisms have been used to remove HABs originating from Japan. From the late 1970s to the early 1980s, natural clay (NC) was used in research on HAB control in the coastal regions of Kagoshima, Japan. However, there were several problems with this method, such as the low HAB organism removal efficiency and the large quantities of clay required [5,6]. NC is a mineral material with a lamellar structure consisting of silicon-oxygen (Si-O) tetrahedra and aluminium-oxygen (Al-O) tetrahedra, in which case lower-valence cations, such as sodium (Na^+^), are prone to replace Al^3+^. This lattice replacement affects the surface electrical properties of the NC particles, making them covered with negative charges in a water environment [7]. Previous studies have shown that the flocculation efficiency of clay for algal organisms is affected by the surface electrical properties, lamellar structure, particle size, and shape (such as tubular or spherical) of the clay particles [6,8,9]. Based on Derjaguin-Landau-Verwey-Overbeek (DLVO) theory, focused on van der Waals forces and electrostatic forces, Yu et al. (1994) established a theoretical model of the mechanism by which clay particles flocculate algal organisms and proposed modified clay (MC) technology [7]. MC technology greatly improved the algal removal efficiency, and the dosing requirement decreased from 110–400 t km^−2^ to 4–10 t km^−2^ [4]. With a number of advantages, including a lack of toxicity, low cost, and high efficiency, MC technology has become one of the most common methods for HAB mitigation and has been applied in various aquatic systems worldwide [4,10]. It was found that the main reason for the high algal organism flocculation efficiency of MC is that the surface of refined clay particles becomes positive in a water environment, followed by an increased effective radius and increased electrostatic force and van der Waals effects for HAB organisms, strengthening the bridging effects between clay particles and HAB organisms [4].

Interestingly, on the basis of field experience, a 70–80% removal efficiency is sufficient for MC to control HABs; that is, even when the amount of residual biomass (20–30% of the initial HAB biomass) remains at the bloom level, further growth or a continued bloom have rarely been observed [4]. What happened to these residual cells? This issue has received much attention in recent years. At the physiological and biochemical levels, Liu et al. found that, after treatment with MC, residual cells of *Amphidinium carterae* and *Karenia mikimotoi* experienced peroxidation, the antioxidant system was stimulated, and photosynthesis was blocked [11,12]. Ji et al. and Zhu et al. found that programmed cell death (PCD) occurred in residual cells of *Prorocentrum donghaiense* and *Aureococcus anophagefferens* [13,14]. From a molecular perspective, Zhu et al. found that transcriptional expression changed in *A. anophagefferens* residual cells that experienced oxidative stress and disturbances of physiological processes [15]. Why do these physiological responses occur in residual algal cells? What properties of MC affect the growth of residual algal cells? To explore these scientific issues and clarify the mechanism by which MC mitigates HABs, the typical HAB (brown tide) organism *A. anophagefferens* [16] was used as the model organism in this study. The investigation considered cell morphology, with a focus on the cell shape, cell size, cell membrane permeability, and cytoplasmic granularity changes in residual cells treatment with NC and MC, as well as the physical and chemical properties of NC and MC particles, such as particle size, surface potential, Young’s modulus, adhesion, and absorption sites, to reveal the mechanism by which MC inhibits the growth of residual cells and prevents a second outbreak of HABs.

## 2. Materials and Methods

### 2.1. Materials

#### 2.1.1. NC and MC

The NC used in these experiments was kaolinite collected in Jiangsu Province, China. The physical and chemical properties of the clay, containing 75.0% SiO_2_, have been described by Liu et al. [17,18]. The modifier was polyaluminium chloride (PAC), obtained from Guangfu Fine Chemical (Tianjin, China). The MC preparation method was reported by Yu et al. [8]. The specific process was as follows: kaolinite and PAC were mixed (5:1) in a reactor, and the mixture was diluted in deionized water, stirred thoroughly, and aged for approximately 24 h at room temperature; then, the mixture was subjected to filter pressing, dehydration, and desiccation. The MC stock suspensions were prepared by diluting the MC mixture in deionized water to a concentration of 25 g L^−1^.

#### 2.1.2. Algal Strain and Culture

*A. anophagefferens* strain CCMP 1984 (obtained from the US National Center for Marine Algae and Microbiota (East Boothbay, ME, USA)) was used in all the culture experiments. Cells were grown in 5-L flasks cultured with L1 medium with no Si [19], diluted in natural seawater filtered with a 0.22 μm mixed-fiber membrane (Xinya, China), and autoclaved. All cultures were maintained at 19 ± 1 °C under a 12:12 h light:dark cycle with cool-white fluorescent light at 50 μmol photons m^−2^ s^−1^ until the algal cells had grown to the mid-exponential phase.

### 2.2. Experimental Methods

#### 2.2.1. Algal Cell Density Determination

Liu et al. found a positive correlation between *A. anophagefferens* cell density and in vivo fluorescence intensity (RFU) [17]. In addition, we found that the chlorophyll fluorescence values of residual cells for the two experimental groups changed nonsignificantly. Therefore, RFU, as measured with a Trilogy fluorometer (Turner Designs, San Jose, CA, USA), was used to represent the cell density of the cultured algae and calculate the HAB removal efficiency in this study.

#### 2.2.2. HAB Removal Experiments

The mid-exponential phase *A. anophagefferens* cultures were mixed thoroughly, divided into three groups, and deposited in 1-L volumetric cylinders. The control group (group 1) did not receive any NC or MC treatment, group 2 received NC treatment, and group 3 received MC treatment. All groups were treated in triplicate in this study. NC and MC stock suspensions were added to the group 2 and group 3 algal cultures to final concentrations of 2.0 g L^−1^ and 0.5 g L^−1^, respectively. Then, all cultures of the experimental groups were placed under the incubation conditions described in Section 2.1.2 and allowed to stand and flocculate until settling was complete after 3 h. The removal efficiency was used to represent the flocculated biomass of the algae. The residual cells that were still suspended were immediately transferred to new containers for further study. At the same time, the RFU for all groups was measured, and the removal efficiency was calculated as follows [18].

(1) $$\mathrm{Removal}\;\mathrm{efficiency}\;\left(\%\right)=\left(1-\frac{final\;fluorescence\;of\;treatment}{final\;fluorescence\;of\;control}\right)\times 100\%.$$

Given the effectiveness of MC for controlling HABs and our desire to collect adequate samples, this research did not aim at a high removal efficiency (100%), and the residual cells accounted for 20–30% of the initial biomass (100%—removal efficiency).

#### 2.2.3. Cell Shape and Size Change Observations and Cytoplasmic Granularity Determination

The cell morphological features of *A. anophagefferens* include a characteristic coccoid or ellipsoidal shape with a diameter of approximately 2 μm, with no cell wall and no flagellum [20]. Due to the small size and simple morphology, it is difficult to identify the morphological differences among algae cells with ordinary light microscopy; however, with the advantage of high resolution, scanning electron microscopy (SEM) can be used to observe the surface morphology of microalgae precisely [21,22]. Therefore, SEM was used to observe *A. anophagefferens* cells and identify the morphological differences between normal cultured cells and residual cells treated with NC or MC. Cells in each group were collected individually at 24 h, 72 h, and 120 h after clay treatment and then fixed with glutaraldehyde for 30 min. Subsequently, all samples were dehydrated with 30%, 50%, 70%, 80%, 90%, and 100% alcohol for 10 min. Finally, isoamyl acetate was used to completely replace the residual alcohol. All samples were dried at the CO_2_ gas-liquid equilibrium point using an HCP-2 (Hitachi, Japan). After the samples were coated with gold, cell morphological changes were observed with a SEM (S-3400N, Hitachi, Japan).

Additionally, through the use of light scattering, fluorescence, and absorbance measurements of stained and unstained cells, a very wide range of cellular parameters can be measured with flow cytometry. When the laser beam impacts a cell, the excitation light is scattered both forward and sideways. The forward-scattered light (FSC-H) provides information on the size of the cells and can be detected without further manipulation. The sideways-scattered light (SSC-H) can reflect cell characteristics on cytoplasmic granularity and cell size [23]. Therefore, this study applied flow cytometry (FACS Calibur, BD, Franklin Lakes, NJ, USA) to measure the cell size and cytoplasmic granularity of normal and residual cells.

#### 2.2.4. Membrane Permeability Determination

SYTOX Green is a high-affinity nucleic acid stain that can penetrate only into cells with compromised cell membranes, fluoresces bright green when excited at 450–490 n [24,25], and can be measured by flow cytometry through a fluorescence 1 panel (FL1). Therefore, this staining technique allows ecotoxicity assessments at the single-cell level. Normal and residual algal cells were directly obtained from the cultured flasks, individually stained with SYTOX Green at a final concentration of 1.0 μmol L^−1^ according to the reagent instructions and the literature, mixed well, and incubated at 19 ± 1 °C in the dark for 10 min [26,27]. Algal cells heated in a water bath (60 °C, 30 min) served as the positive control. Chlorophyll fluorescence emitted by algae cells was measured by a fluorescence 3 panel (FL3).

#### 2.2.5. Characteristics of NC and MC

In addition to particle size, shape (tube-shaped or spherical), surface electrical properties, and environmental pH, it has been reported that absorption sites play an important role in clay flocculation [28,29,30]. Recently, some properties of the particle surface, such as adhesion and rigidity, which also affect the flocculation efficiency, are also receiving much attention [31,32,33]. In this study, the particle size of NC and MC was assessed using laser granulometry (Mastersizer A3000, Malvern, UK); the surface potential was measured by a Zeta Sizer (Zetasizer nano ZS, Malvern, UK); and the lamellar structure, adhesion and Young’s modulus were determined by atomic force microscopy (AFM) (Resolve, Bruker, Billerica, MA, USA) in peak force-QNM-air mode.

## 3. Results and Discussion

### 3.1. Cell Growth in Response to MC

**Figure 1:**
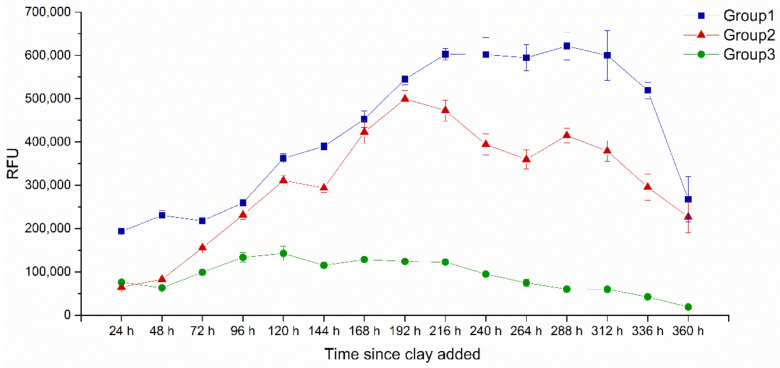
The change in cell growth under the impact of NC or MC.

The removal efficiencies of group 2 and group 3 were 72.1% and 71.3%, respectively, after treatment with NC or MC. At this removal efficiency, it was not only MC could control HABs effectively but also supplied enough cells for further study. Then, the growth of *A. anophagefferens* in the three groups was monitored for 360 h after NC or MC addition (Figure 1). The density of algal cells in group 1 continuously increased for 216 h, then maintained for 96 h in a plateau stage, and began to decrease at 336 h. For group 2, the density of residual cells continuously increased for 192 h, reaching 80.3% of the highest cell density of group 1. Although there was no obvious plateau in group 2, the cell density decreased slowly. At 360 h, the cell density in group 2 was 84.7% that in group 1. Interestingly, the cell density of group 3 continuously increased for 120 h and reached only 23.0% of the highest density of group 1. After that, the density began to decrease and reached 1.1% that in group 1 at 360 h. The results showed that, when the removal efficiency reached approximately 70%, NC caused a weak inhibition effect on the growth of residual cells, and the bloom could burst again due to the rapid growth rate and large biomass; in contrast, MC could effectively inhibit the growth of residual cells and block a second bloom.

### 3.2. Changes in Cell Shape and Size of Residual A. anophagefferens

**Figure 2:**
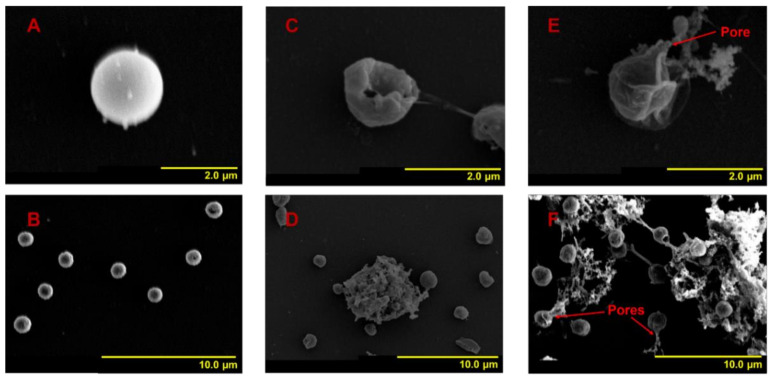
Changes in cell shape features of residual *A. anophagefferens* (CCMP 1984) after treatment with NC or MC for 24 h. (**A**,**B**) Normal cells (group 1); (**C**,**D**) residual cells treated with NC (group 2); (**E**,**F**) residual cells treated with MC (group 3).

**Figure 3:**
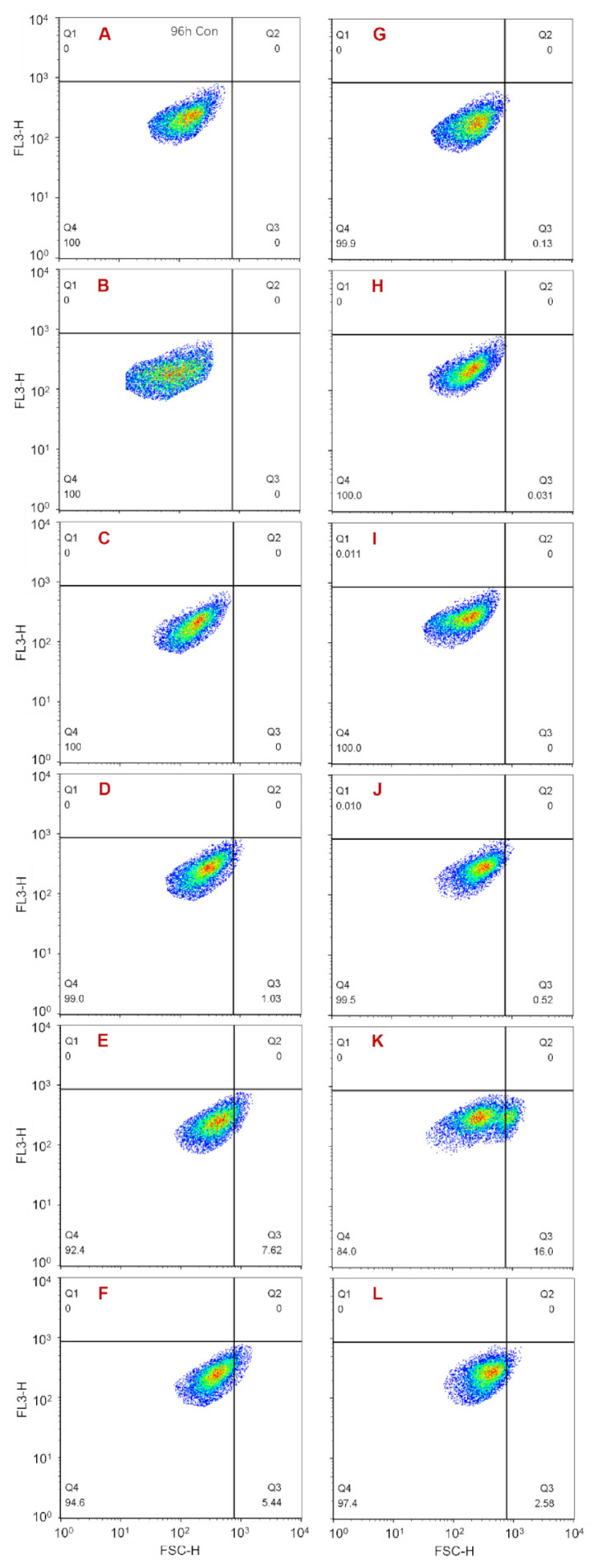
Changes in the cell size of residual *A. anophagefferens* (CCMP 1984) within 96 h after treatment with NC or MC. (**A**,**G**) Normal cells measured at 3 h and 96 h, respectively (group 1); (**B**–**F**) residual cells treated with NC measured at 3 h, 24 h, 48 h, 72 h, and 96 h, respectively (group 2); (**H**–**L**) residual cells treated with MC measured at 3 h, 24 h, 48 h, 72 h, and 96 h, respectively (group 3).

Using SEM, the cell shape characteristics of the three groups were recorded within 120 h after NC or MC addition. Flow cytometry was applied to record the size differences in the three groups of algal cells within 96 h. At 24 h, *A. anophagefferens* cells in group 1 were coccoid or ellipsoidal in shape with a diameter of approximately 2 μm (Figure 2A,B). These are normal morphological characteristics for *A. anophagefferens,* which is consistent with previous reports [20,34]. In addition, the peripheries of some group 1 cells were fuzzy, and the surfaces were not smooth but had substances attached (Figure 2A,B). It has been reported that *A. anophagefferens* covered with an extracellular polysaccharide (EPS) sheath presents high viscosity, which makes it adhere to the gills of filter-feeding animals when forming blooms, thus inhibiting filter feeding and blocking respiration [35,36]. Therefore, these fuzzy cell peripheries are caused by the presence of EPS. After treatment with NC for 24 h, most of the residual cells were still spherical or ellipsoidal, some residual cells showed irregular shapes, and some residual cells were broken and were surrounded with fewer impurities; however, nearly all the residual cells had clear peripheries (Figure 2C,D). After treatment with MC for 24 h, the proportion of irregularly shaped and broken residual cells was larger than that in group 2. In addition, numerous impurities surrounded the broken cells, and cross-linking occurred between cells (Figure 2E,F). All the residual cells in group 3 had clear peripheries, similar to those in group 2. Comparing the broken cells in group 2 to those in group 3 revealed two different types of breakage. In group 2, broken cells suffered a large area of membrane depression and rupture, while, in group 3, the broken cells burst, and the cytoplasmic contents flowed out through pores in the cell membrane. In both group 2 and group 3, nearly all the residual cell peripheries became clear, illustrating that NC and MC can decrease EPS, in accordance with the report by Ren et al. [37]. It should be noted that *A. anophagefferens* does not have a cell wall, and the EPS sheath is an important barrier. The loss of the EPS protective layer causes the entire cell (protoplast) to be directly exposed to the environment, threatening survival [38,39]. At this time, the volume of residual cells in both experimental groups did not change significantly (Figure 3A,B,H).

**Figure 4:**
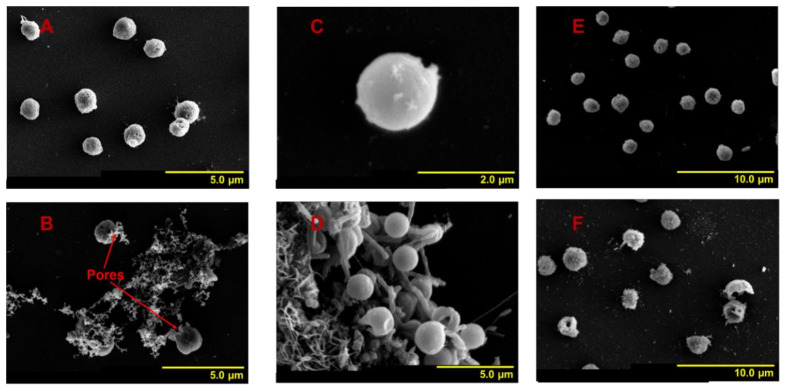
Changes in cell shape features of residual *A. anophagefferens* (CCMP 1984) after treatment with NC or MC for 72 h and 120 h. (**A**,**E**) Residual cells treated with NC measured at 72 h and 120 h, respectively (group 2); (**B**,**F**) residual cells treated with MC measured at 72 h and 120 h, respectively (group 3); (**C**,**D**) normal cells measured at 120 h (group 1).

At 72 h, the morphological features of the algal cells in group 1 were consistent with those at 24 h. In group 2, almost all residual cells remained spherical or ellipsoidal, with intact cell boundaries, and were merely broken (Figure 4A). In group 3, there were still a large number of irregularly shaped and broken cells with cytoplasmic contents released from the membrane pores (Figure 4B). At this time, the volume of cells in group 1 was equal to the original volume. In group 2, the size of residual cells increased slightly (Figure 3E). Notably, in group 3, a sub-cell population characterized with significantly larger cell volume appeared (*p* < 0.05), equivalent to 25.6% of the total number of cells in group 2 (Figure 3K). At 96 h, the volume of cells in group 1 remained consistent with the original value (Figure 3G). In group 2, the volume of residual cells increased slightly (Figure 3F). In group 3, the volume of residual cells also increased slightly, similar to the results in group 2, and the larger cell volume sub-cell population that occurred at 72 h disappeared (Figure 3L). At 120 h, cells in group 1 maintained their morphological features, while intercellular connectivity enhanced the formation of cell clusters, and many bacteria were generated, indicating that the viscosity of the cell surface and the organic matter of the culture system were increased (Figure 4C,D). In group 2, almost all residual cells remained spherical or ellipsoidal with intact cell boundaries (Figure 4E). However, there were various shapes of residual cells in group 3, and their volumes were significantly larger than those in group 1 and group 2 (Figure 4F). Moreover, some broken cells still existed (Figure 4F). Additionally, during the whole monitoring period, residual cells in the two experimental groups had clear peripheries, and few bacteria appeared in the culture systems, indicating that both NC and MC could reduce the EPS surrounding the cell surface and keep the organic matter at a low level for a long time (Figure 4).

Based on the results described above, it could be concluded that: (1) NC and MC could reduce EPS surrounding the cell surface and keep organic matter at a low level for a long time. (2) NC and MC could cause residual cells to form various shapes or even to burst. Both the existence time and the proportion of irregularly shaped cells and broken cells were smaller in group 2, and the latter occurred only at 24 h; however, in group 3, broken cells existed throughout the whole monitoring period. Moreover, there were two different causes of cell breakage in group 2 and group 3. One mechanism is the depression of a large area of the cell membrane, and the other mechanism is the formation of pores in the cell membrane, followed by the cytoplasmic contents outflowing. (3) The cell volume of residual cells with MC treatment increased significantly at 72 h and 120 h; however, this phenomenon was not recorded in group 2. These results indicated that the damage to residual cells caused by MC was greater than that caused by NC, the cell growth inhibition mechanisms caused by NC and MC were different, and the latter mechanism was more complex.

### 3.3. Changes in Cell Membrane Permeability for Residual A. anophagefferens

**Figure 5:**
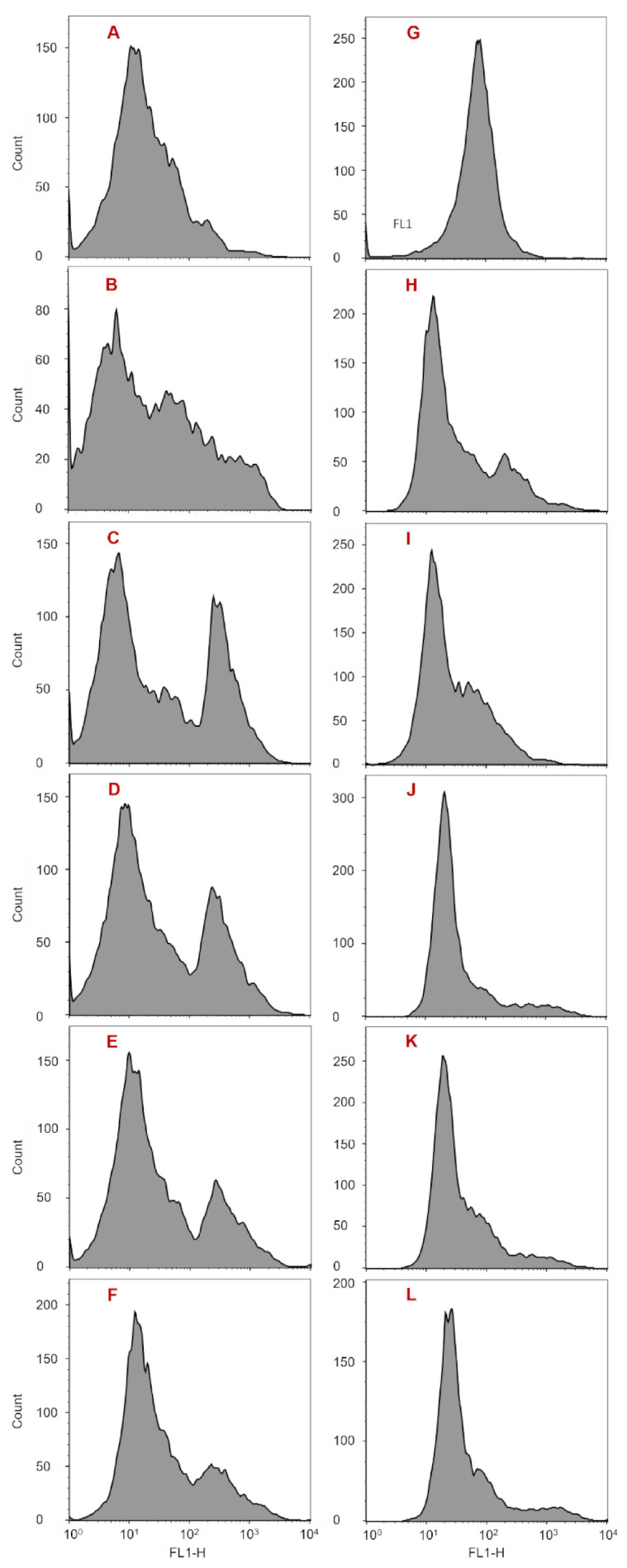
Cell death of residual cells within 96 h after treatment with NC or MC (SYTOX Green staining). (**A**) Normal cells measured at 3 h (group 1); (**B**–**F**) residual cells treated with NC measured at 3 h, 24 h, 48 h, 72 h, and 96 h, respectively (group 2); (**H**–**L**) residual cells treated with MC measured at 3 h, 24 h, 48 h, 72 h, and 96 h, respectively (group 3); (**G**) SYTOX Green-positive cells.

After staining with SYTOX Green, the cell membrane permeability of the three groups was recorded using flow cytometry. From another perspective, some changes in membrane permeability mean cell death, so the SYTOX Green-positive cells were considered dead. The results showed that, during the 96 h monitoring period, in group 2, a SYTOX Green-positive cell population was present throughout and was maintained at 7.2–9.8% (Figure 5B–F). In contrast, in group 3, a positive cell population occurred only at 3 h and accounted for 3.8% of the total cells measured in the group (Figure 5H–L). The morphological study results in Section 3.2 showed that a certain amount of broken cells existed throughout the whole monitoring period in group 3 but occurred only at 24 h in group 2. More importantly, there were some pores within the cell membranes of group 3 residual cells which caused the cytoplasmic contents to flow out and made it difficult to maintain cell integrity. Combining these results it could be illustrated that dead residual cells caused by NC could exist in an intact cell state for a period of time, while dead residual cells caused by MC burst until lysing and were not present at intact cells. The results further indicated that the damage to residual cells caused by MC was greater than that caused by NC.

### 3.4. Changes in Cytoplasmic Granularity for Residual A. anophagefferens

**Figure 6:**
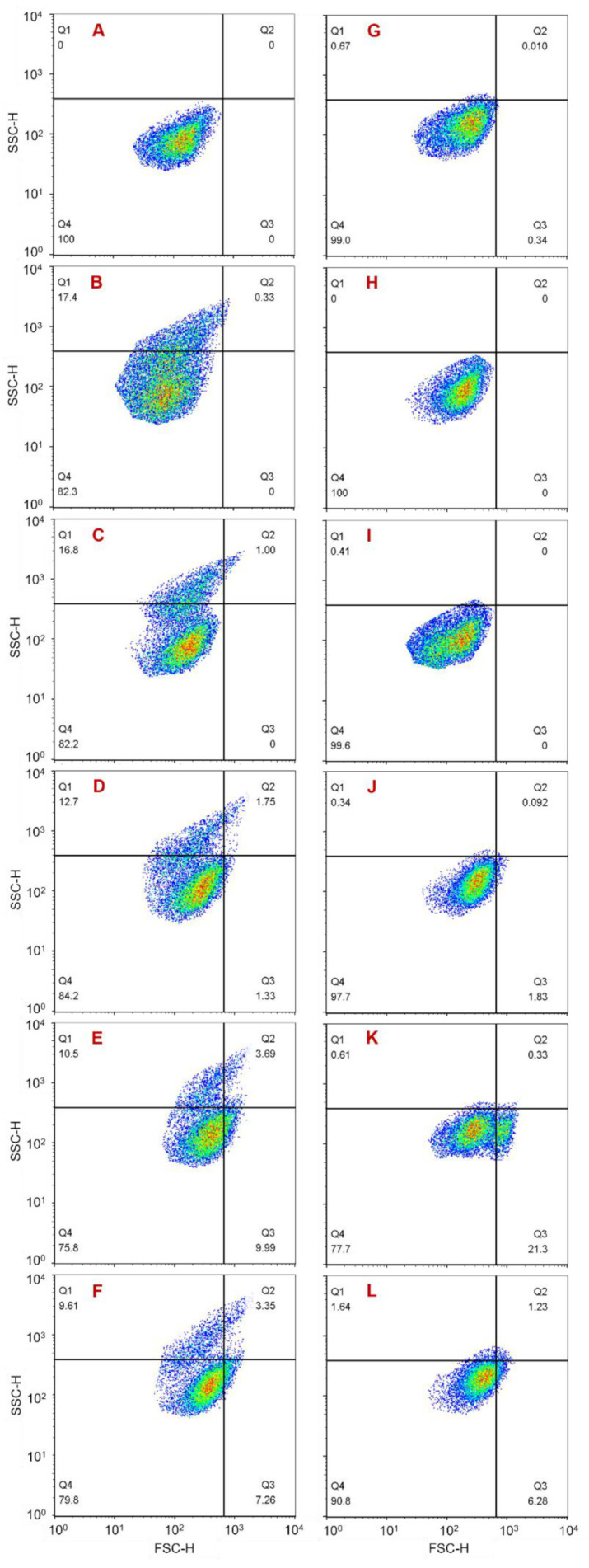
Changes in cytoplasmic granularity of residual cells within 96 h after treatment with NC or MC. (**A**,**G**) Normal cells measured at 3 h and 96 h, respectively (group 1); (**B**–**F**) residual cells treated with NC measured at 3 h, 24 h, 48 h, 72 h, and 96 h, respectively (group 2); (**H**–**L**) residual cells treated with MC measured at 3 h, 24 h, 48 h, 72 h, and 96 h, respectively (group 3).

The results obtained from flow cytometry showed that during the 96 h monitoring period, in group 2, a population with an elevated SSC-H value was present throughout, although its proportion decreased gradually from 31.0% to 16.1% (Figure 6B–F). In contrast, there was no such cell population in group 1 and group 3 (Figure 6A,G–L). In the measuring process, the SSC-H value was affected by cytoplasmic granularity, cell size, and cell shape. Within the 96 h monitoring period in this study, there was a proportion of irregularly shaped residual cells in both group 2 and group 3, and the size of residual cells in the two groups increased gradually over time (Figure 2, Figure 3 and Figure 4). However, there was no cell population with an elevated SSC-H value in group 3 (Figure 6H–L). Therefore, it could be inferred that the presence of the cell population with an elevated SSC-H value in group 2 was due to increased cytoplasmic granularity. This indicates that there was a cell population with increased cytoplasmic granularity in group 2 but not in group 3. This phenomenon is similar to the SYTOX Green-positive cell population that existed only in group 2. The reason is that the dead residual cells caused by NC could exist in an intact cell state for a period of time, while the dead residual cells caused by MC burst until lysing were not present as intact cells. These results further indicated that the inhibition mechanisms caused by NC and MC to residual cell growth were different, and MC resulted in greater damage.

### 3.5. The Mechanism by Which MC Controls HABs from a Cell Morphology Perspective

Why can NC and MC cause distinct forms of damage to residual cells and only MC effectively inhibit the growth of residual cells, preventing a second bloom? The main interaction between algal cells and clay particles in culture systems is collision [4]. Collision that makes algal cells and clay particles combine to form flocs, leading to sedimentation, is also known as effective collision. This is the way that clay effectively reduces HAB biomass in a short time. Conversely, collision that does not result in sedimentation is known as ineffective collision. Although ineffective collision cannot directly reduce HAB biomass, it can cause damage to algal cells, which is the main way clay inhibits cell growth [15]. The residual cells employed in this study included cells subjected to ineffective collision. After modification with PAC, the physical and chemical properties of the clay particles changed, which not only improved the effective collision efficiency but also greatly increased the damage to residual cells caused by ineffective collision. Therefore, MC can effectively inhibit the growth of residual cells, preventing the continuation and secondary outbreaks of HABs.

**Figure 7:**
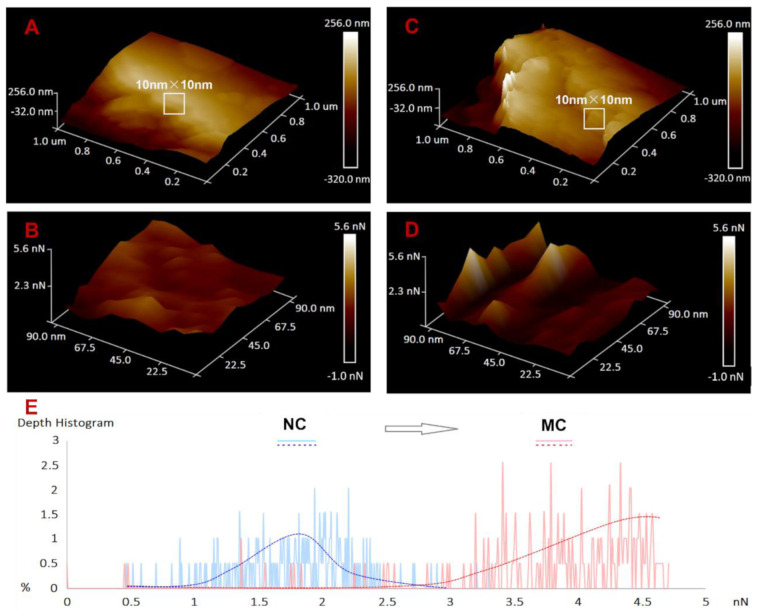
NC and MC morphology and adhesion distribution map measured by atomic force microscopy. (**A**) Three-dimensional image for morphology of NC; (**B**) three-dimensional image for morphology of MC; (**C**) three-dimensional image for adhesion distribution of NC; (**D**) three-dimensional image for adhesion distribution of MC; (**E**) the adhesion values distribution for NC and MC.

Combining the results and discussion described in Section 3.2, Section 3.3 and Section 3.4, it could be concluded that, from a cell morphology perspective, NC caused various types of damage to residual cells, such as cell deformation, cell breakage, decreased EPS, increased cell membrane permeability, and increased cytoplasmic granularity. However, because these damages are caused by physical collisions between clay particles and algae cells, the inhibition to residual cell growth is weak, and cells can still grow rapidly, reaching 80.3% of the highest cell density of group 1. After modification, clay particles not only exhibit reversed surface electrical properties and increased clay lamellar spacing but also decreased hardness, increased adhesion chains, improved adhesion, and increased absorption sites (Figure 7). These changes in the physical and chemical properties of clay particles not only enhanced the physical damage to residual cells but also added chemical and electrochemical damage, which resulted in residual cell injury and growth inhibition, followed by HAB dissipation.

**Figure 8:**
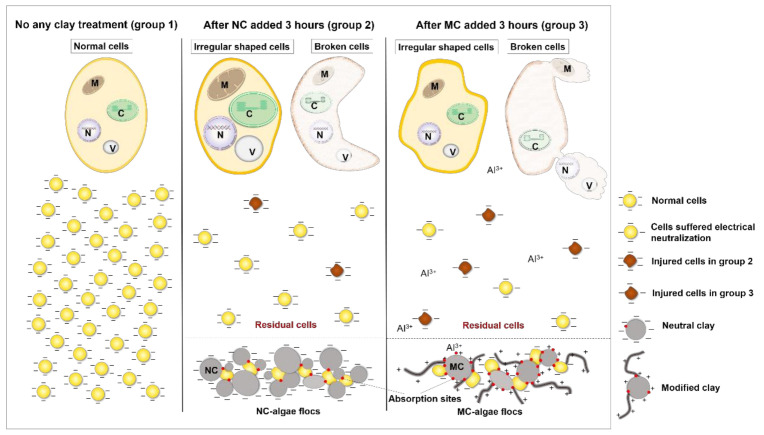
Schematic showing the proposed cell morphology changes for HAB controlled by NC and MC. In group 2, there were some irregularly shaped and cytoplasmic granularity increase residual cells, and broken cells suffered a large area of membrane depression. In group 3, the proportion of irregularly shaped and broken cells is larger than that in group 2, and broken cells with cytoplasmic contents released from the membrane pores. C, chloroplast; M, mitochondrion; N, nucleus; V, vacuole.

First, after modification, the surface potential of clay particles increased from −13.10 mV to +11.30 mV. This change in surface electricity contributes to electrical neutralization when clay particles contact algal cells covered by negative charges. This electrochemical effect shocked residual cells and disordered their physiological processes [15]. In this study, this effect was reflected in cell membrane perforation, as shown in Figure 2 and Figure 4, with cytoplasmic contents flowing out through pores within the cell membrane and cell lysis. Moreover, this effect remained functional in the long term, illustrating that electrical neutralization is one of the main reasons why MC could effectively inhibit cell growth and lead to severe cell breakage. Second, because the clay particle surface became fluffier (lamellar spacing increased) and softer (Young’s modulus decreased from 199 MPa to 99 MPa), adhesion roughly doubled (increased from 2.10 nN to 3.82 nN), and the number of absorption sites with peak force increased (Figure 7B,D), the interaction time and force between clay particles and algae cells were improved, causing greater damage to algal cells and leading to more irregularly shaped and broken cells. This is another important reason why MC blocks HABs. Finally, when the macromolecular modifier PAC combined with the clay particles, long chain-type “tentacles” grew on the clay particle surfaces, causing chemical damage to residual cells besides physical collision. In addition, when MC is added to cell cultures, active Al^3+^ can dissociate into the water, enhancing the chemical damage to residual cells [40]. This chemical factor also plays an important role in the blocking of HABs by MC. Under these effects of MC, residual cells present chemical damage characteristics, such as swelling and membrane perforation [41]. Therefore, after modification, the occurrence of chemical and electrochemical effects and the enhancement of physical collision make MC effectively inhibit the growth of residual cells and control HABs (Figure 8).

## 4. Conclusions

After modification, some physical and chemical properties of clay particles are changed. Therefore, apart from enhancing physical collisions with residual cells, MC caused chemical and electrochemical effects on residual cells, while NC only caused physical effects. In addition, only residual cells treatment with MC presented chemical damage characteristics with cytoplasmic contents flowing out through pores in the cell membrane. Under the effects of MC, severe cell deformation and chemical cell breakage lead to cell growth inhibition. This study explained why MC could inhibit the growth of residual cells and control HABs effectively from the perspective of cell morphology, optimized the mechanism of MC controlling HABs, and had important scientific significance for further research and development efficient MC species.

## Data Availability

Data are available upon request; please contact the contributing authors.
